# Identification of prognostic gene signature associated with microenvironment of lung adenocarcinoma

**DOI:** 10.7717/peerj.8128

**Published:** 2019-11-29

**Authors:** Cheng Yue, Hongtao Ma, Yubai Zhou

**Affiliations:** Department of Biotechnology, College of Life Science & Bioengineering, Beijing University of Technology, Beijing, China

**Keywords:** Lung adenocarcinoma, Tumor microenvironment, Prognosis, Gene signature, Bioinformatical analysis

## Abstract

**Background:**

Lung cancer has the highest morbidity and mortality worldwide, and lung adenocarcinoma (LADC) is the most common pathological subtype. Accumulating evidence suggests the tumor microenvironment (TME) is correlated with the tumor progress and the patient’s outcome. As the major components of TME, the tumor-infiltrated immune cells and stromal cells have attracted more and more attention. In this study, differentially expressed immune and stromal signature genes were used to construct a TME-related prognostic model for predicting the outcomes of LADC patients.

**Methods:**

The expression profiles of LADC samples with clinical information were obtained from The Cancer Genome Atlas (TCGA) and Gene Expression Omnibus (GEO). The differentially expressed genes (DEGs) related to the TME of LADC were identified using TCGA dataset by Wilcoxon rank sum test. The prognostic effects of TME-related DEGs were analyzed using univariate Cox regression. Then, the least absolute shrinkage and selection operator (LASSO) regression was performed to reduce the overfit and the number of genes for further analysis. Next, the prognostic model was constructed by step multivariate Cox regression and risk score of each sample was calculated. Then, survival and Receiver Operating Characteristic (ROC) analyses were conducted to validate the model using TCGA and GEO datasets, respectively. The Kyoto Encyclopedia of Genes and Genomes analysis of gene signature was performed using Gene Set Enrichment Analysis (GSEA). Finally, the overall immune status, tumor purity and the expression profiles of HLA genes of high- and low-risk samples was further analyzed to reveal the potential mechanisms of prognostic effects of the model.

**Results:**

A total of 93 TME-related DEGs were identified, of which 23 DEGs were up-regulated and 70 DEGs were down-regulated. The univariate cox analysis indicated that 23 DEGs has the prognostic effects, the hazard ratio ranged from 0.65 to 1.25 (*p* < 0.05). Then, seven genes were screened out from the 23 DEGs by LASSO regression method and were further analyzed by step multivariate Cox regression. Finally, a three-gene (ADAM12, Bruton Tyrosine Kinase (BTK), ERG) signature was constructed, and ADAM12, BTK can be used as independent prognostic factors. The three-gene signature well stratified the LADC patients in both training (TCGA) and testing (GEO) datasets as high-risk and low-risk groups, the 3-year area under curve (AUC) of ROC curves of three GEO sets were 0.718 (GSE3141), 0.646 (GSE30219) and 0.643 (GSE50081). The GSEA analysis indicated that highly expressed ADAM12, BTK, ERG mainly correlated with the activation of pathways involving in focal adhesion, immune regulation. The immune analysis indicated that the low-risk group has more immune activities and higher expression of HLA genes than that of the high-risk group. In sum, we identified and constructed a three TME-related DEGs signature, which could be used to predict the prognosis of LADC patients.

## Introduction

Lung cancer is the deadliest malignant disease in the world with about two million new cases and 1.8 million deaths each year (Bray et al., 2018). According to histological examination, lung cancer can be divided into small cell lung cancer (SCLC, ∼20%) and non-small cell lung cancer (NSCLC, ∼80%) (Leung et al., 2016; Mendes et al., 2015). The NSCLC can be further classified into lung squamous cell carcinoma, lung adenocarcinoma (LADC) and large cell carcinoma, and LADC is the most common subtype of lung cancer (Pao & Girard, 2011; Sullivan, Minna & Shay, 2010). Different subtypes of lung cancer are quite different in terms of molecular characteristics and treatments (Lockwood et al., 2012). In the past decades, extensive genomic studies have identified several high frequent genetic alternations in LADC, such as EGFR, KRAS mutations and ALK rearrangements, which may be involved in the tumorigenesis and progress of LADC, and lead to the development of targeted drugs of EGFR tyrosine kinase inhibitor represented by gefitinib (Herbst, Morgensztern & Boshoff, 2018). With the advance in surgery and chemoradiotherapy, as well as the introduction of targeted drugs and immunotherapy, great progress has been made in the treatment of lung cancer. However, the prognosis of lung cancer is still dismal. Its 5-year overall survival (OS) rate remains less than 20% (Chen et al., 2014; Ettinger et al., 2013).

Although the genetic and epigenetic changes in tumor cells are crucial to the oncogenesis and progress of tumors, accumulating evidence shows that the interaction among the tumor cells and its surrounding normal cells also plays an important role (Quail & Joyce, 2013). The tumor microenvironment (TME) is a complex network composed of tumor cells, mesenchymal stem cells, fibroblast cells, endothelial cells, inflammatory cells and extracellular matrix. As the major cellular components (CCs) of the TME, the immune infiltrating cells and stromal cells are getting more and more attention. Evaluation of the status of these two types of cells in TME will contribute to more accurate diagnosis and prognosis evaluation of tumor patients. Currently, a variety of bioinformatics tools are available to assess the distribution of immune and stromal cells in the TME (Carter et al., 2012; Yoshihara et al., 2013). Among them, the Estimation of STromal and Immune cells in MAlignant Tumour tissues using Expression data (ESTIMATE) method has been successfully applied to the quantitative analysis of TME of various tumors, and its effectiveness has been proved (Alonso et al., 2017; Jia et al., 2018; Priedigkeit et al., 2017; Shah et al., 2017).

The ESTIMATE package defines a set of TME-related genes which is comprised of immune and stromal signature genes. In this study, the differentially expressed TME-related genes between LADC and normal samples were identified using the LADC transcriptome expression data from The Cancer Genome Atlas (TCGA) database. Next, a three-gene signature was constructed and evaluated using independent database and its potential prognostic mechanisms were further analyzed. In conclusion, a three-gene signature associated with LADC TME was constructed, which can be used to predict the OS of LADC patients.

## Materials and Methods

### Data source and preprocessing

Fragments per Kilobase Million (FPKM) normalized expression profile data of LADC samples were downloaded from TCGA database using GDC data transfer tool and summarized into an expression matrix. The ensemble ids were converted into gene symbols according to the annotation file (Homo_sapiens.GRCh38.95.CRH.GTF). Then the xml formatted clinical information of LADC patients was downloaded and merged into a single matrix for further analysis. Three Gene Expression Omnibus (GEO) datasets, GSE3141, GSE30219 and GSE50081, which contained the microarray-based expression data of LADC patients and associated clinical information were downloaded from GEO website (https://www.ncbi.nlm.nih.gov/geo/) via GEOquery package in R software (Davis & Meltzer, 2007). All GEO datasets used GPL570 platform. The probe ids were converted into gene symbols according to related annotation file, and for multiple probes corresponding to the same gene, the average expression value was calculated.

### Identification of differentially expressed genes related to LADC tumor microenvironment

ESTIMATE is an algorithm that infers the infiltration situation of immune cells and stromal cells in tumor tissue according to the transcriptome data of TME-related genes which contain a set of immune and stromal signature genes (Yoshihara et al., 2013). The expression data of these TME-related genes were extracted from TCGA LUAD dataset. Differentially expressed TME-related genes between LADC and normal samples were screened using Wilcoxon rank sum test. The False Discovery Rate (FDR) in multiple comparisons was controlled using Benjamini–Hochberg procedure (Benjamini & Hochberg, 1995). The screening criteria were |log2(Fold Change)| > 1 and FDR < 0.05.

### Functional enrichment, KEGG and PPI network analysis

The clusterProfiler package was used for Gene Ontology (GO) enrichment and Kyoto Encyclopedia of Genes and Genomes (KEGG) pathway analysis, and *p*. adjust (FDR) < 0.05 was considered statistically significant (Yu et al., 2012). Protein–protein (PPI) interactions network can visualize the patterns of molecular interactions and help to explain the mechanisms underlying phenotypes. To further explore the interactions among TME-related differentially expressed genes (DEGs), PPI network analysis was performed using the online database STRING with interaction score of 0.4 as the threshold (https://string-db.org/) (Szklarczyk et al., 2019).

### The construction of LADC TME-related prognostic model

After removing the patients whose survival time was NA, the resulting 458 LADC patients in TCGA LUAD dataset were included in the Cox regression analysis. The univariate Cox model was used to determine the relationship between TME associated DEGs expression and OS. The *p* < 0.05 was considered statistically significant. The key genes were selected from the significant DEGs in the univariate analysis using least absolute shrinkage and selection operator (LASSO) regression analysis via glmnet package in R software (Friedman, Hastie & Tibshirani, 2010). The LASSO regression is a popular method for variable selection in fitting high-dimension generalized linear model, which can get a more refined model by constructing a penalty function to reduce the variable numbers and effectively avoid overfitting. Then, the selected key genes were included in multivariate Cox analysis, and risk score formula was constructed according the analysis results.

### Validation of the gene signature for survival prediction in the testing dataset

The predictive performance of the TME-related gene signature was further validated in three GEO datasets. Samples in the testing datasets were divided into high- and low-risk group according to the formula of risk score derived from the training dataset, respectively. Kaplan–Meier (KM) survival analysis and receiver operating characteristic (ROC) curve were used to evaluate the predicting power of the gene signature, and the prognostic performance of other clinicopathological factors was also analyzed.

### Function enrichment analysis of TME-related gene signature

We also identified pathways that were up- and down-regulated when the expression level of TME-related gene signature was changed by gene set enrichment analysis (GSEA 4.0) (Subramanian et al., 2005). The curated KEGG gene set was downloaded from the MSigDB database. Enrichment FDR values were based on 1,000 permutations, and FDR < 0.05 was considered to be statistical significance.

### Evaluation of immune status between high-risk and low-risk groups stratified by prognostic model

To explore the potential relationship between immune system and TME-related gene signature, we analyzed the immune status of the high-risk and low-risk samples. First, using 29 immune signatures, we quantified the immune activities between high-risk and low-risk samples by single-sample gene-set enrichment analysis (ssGSEA) (Hänzelmann, Castelo & Guinney, 2013; He et al., 2018). Then, the ESTIMATE algorithm was used to calculate corresponding immune scores, stromal scores and tumor purities (Yoshihara et al., 2013), and the difference of tumor purities, expression of HLA genes between high-risk and low-risk samples was further analyzed.

## Result

### Identification the TME-associated DEGs in LADC

**Figure 1:**
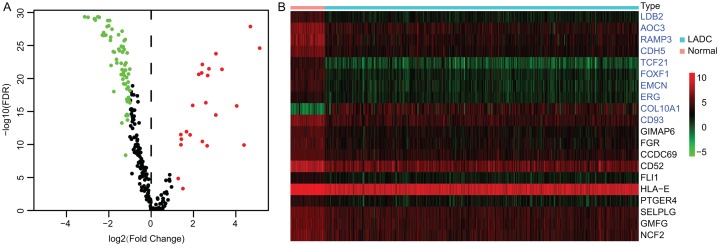
Identification of TME-related DEGs. (A) Volcano plot of TME-related DEGs. |log2(Fold Change)| > 1 and FDR < 0.05 were set as screening criteria. The green, red and black dots represented the down-, up-regulated TME-related DEGs and genes that were not satisfied the screening criteria, respectively. (B) The heatmap of top 10 stromal and immune signature DEGs. The stromal signature DEGs were presented in blue color and immune signature DEGs were in black. TME, Tumor microenvironment; DEGs, Differentially expressed genes; LADC, Lung adenocarcinoma.

Accumulating evidence suggests that tumor progression and patient’s prognosis were associated with the TME. To identify the prognostic LADC TME-related genes, differential expression analysis was conducted. First, the expression profiles of TME associated immune and stromal signature genes were extracted from TCGA LUAD dataset, which contained 141 immune and stromal signature genes respectively (Table S1). Then the DEGs between tumor and normal samples were identified by Wilcoxon rank sum test. Totally, 93 TME-related DEGs were identified, of which 23 DEGs were up-regulated and 70 DEGs were down-regulated (Fig. 1A), and the top 10 immune DEGs and stromal DEGs were presented in heatmap, respectively (Fig. 1B).

### GO, KEGG and PPI analysis of TME-related DEGs

**Figure 2:**
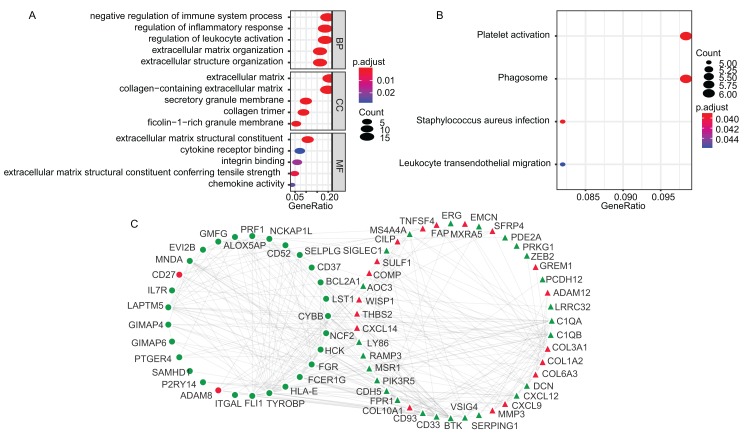
The GO, KEGG and PPI analysis of TME-related DEGs. (A) The GO analysis of TME-related DEGs. (B) The KEGG pathway analysis of TME-related DEGs. (C) The PPI analysis of TME-related DEGs. In the PPI network, red and green represented up- and down-regulation; circle and triangle represented immune and stromal signature DEGs, respectively. BP, Biological Process; CC, Cellular Component; MF, Molecular Function; GO, Gene Ontology; KEGG, Kyoto Encyclopedia of Genes and Genomes; PPI, Protein–Protein Interaction; DEGs, Differentially expressed genes.

The GO enrichment and KEGG pathway analyses of the TME-related DEGs were conducted using clusterProfiler package in R environment (Yu et al., 2012). The DEGs were mainly associated with biological process (BP) of immune responses, such as negative regulation of immune system process (GO:0002683), regulation of inflammatory response (GO:0050727) and regulation of leukocyte activation (GO:0002694) (Fig. 2A). The KEGG analysis showed that the enriched pathways were Platelet activation (hsa04611), *Staphylococcus aureus* infection (hsa05150), Phagosome (hsa04145) and Leukocyte transendothelial migration (hsa04670) (Fig. 2B). The GO terms of the three categories, BP, CC, molecular function (MF) and KEGG results were presented in Table S2 and Table S3, respectively. Using STRING online tool, PPI network of LADC TME-related DEGs was constructed, which contained 93 nodes, 294 edges and average node degree was 6.32 (PPI enrichment *p*-value < 1.0E−16). PPI network also revealed that the stromal signature DEGs had extensively interactions with immune signature DEGs, which is consistent with previous reports that stromal cells and immune cells in the TME work together to form a microenvironment conducive to tumor growth. After removing the disconnected nodes, the PPI network was presented in Fig. 2C.

### Construction and validation of TME-related gene Signature

**Figure 3:**
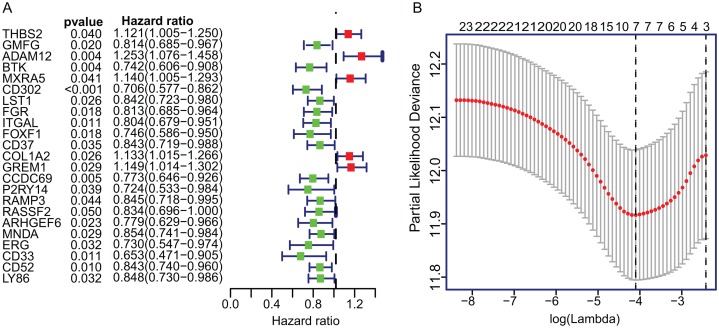
The univariate Cox regression and LASSO analysis for prognostic features screening. (A) The forest plot of 23 prognostic TME-related DEGs screened out by univariate Cox proportional hazards regression. (B) The partial likelihood deviance plot presented the minimum number corresponds to the covariates used for multivariate Cox analysis. DEGs, Differentially expressed genes; LASSO, least absolute shrinkage and selection operator.

To construct the TME-related gene signature, TCGA LUAD dataset (*n* = 458) were included in univariate Cox proportional hazard regression analysis and the resulting 23 significant DEGs (*p* < 0.05) were input in LASSO regression (Fig. 3A). Then, the seven key DEGs were selected to performed the multivariate Cox regression analysis (Fig. 3B). Finally, a prognostic model containing three genes (ADAM12, Bruton Tyrosine Kinase (BTK), ERG) was established to assess the prognosis of each patient as follows:

Risk score = 0.3619 × ADAM12 + (−0.4048) × BTK + (0.2396) × ERG. The detailed information of multivariate Cox regression was presented in Table 1.

**Table 1:** The multivariate Cox regression analysis of key TME-related genes.

Gene	Coef	HR	HR.95L	HR.95H	*p*-value
ADAM12	0.361912	1.436073	1.222805	1.686536	1.02E−05
BTK	−0.40482	0.667096	0.52559	0.846699	0.000875
ERG	−0.23961	0.786932	0.571438	1.083692	0.142195

**Note:**

TME, Tumor microenvironment; HR, Hazard ratio.

**Figure 4:**
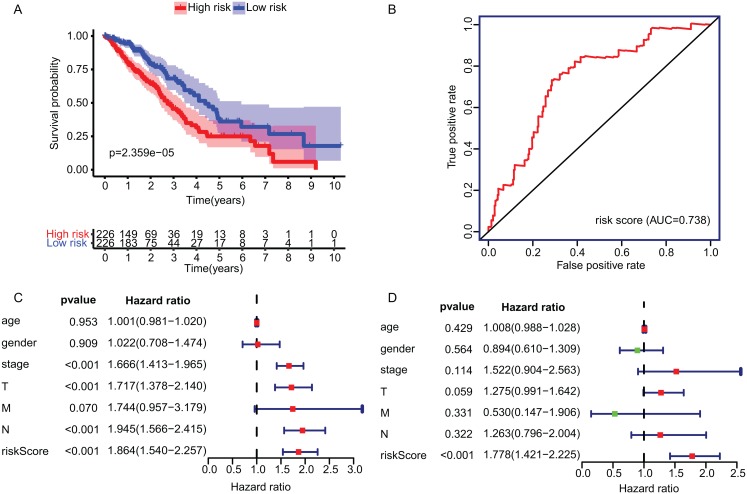
Construction and validation of TME-related gene signature using TCGA dataset. (A) Survival curve of low- and high-risk groups stratified by TME-related gene signature. (B) The ROC analysis of TCGA dataset for survival prediction by TME-related gene signature. (C) The prognostic effect analyses of TME-related gene signature and commonly used prognostic factors using univariate Cox regression model. (D) The independent prognostic effect analyses of TME-related gene signature and commonly used prognostic factors using multivariate Cox regression model. TME, Tumor Microenvironment; TCGA, The Cancer Genome Atlas; ROC, Receiver operating characteristic.

**Figure 5:**
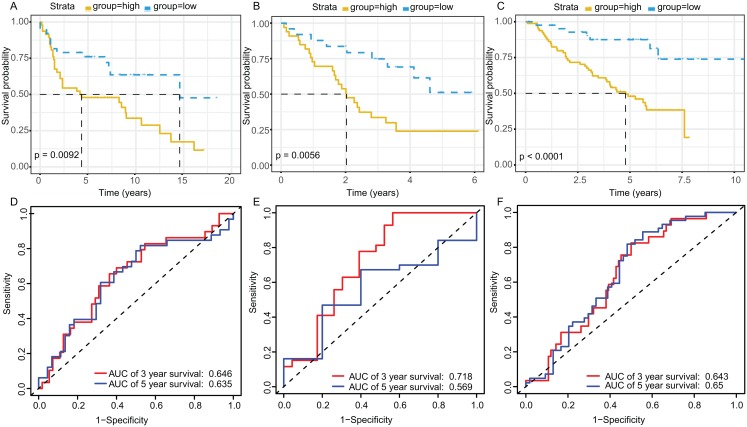
Survival and ROC curves for the three-gene signature in three GEO testing datasets. Kaplan–Meier survival curves showing overall survival outcomes of GSE30219 (A), GSE3141 (B) and GSE50081 (C) according to relative high-risk and low-risk patients. The ROC analysis of GSE30219 (D), GSE3141 (E) and GSE50081 (F) for survival prediction by the three-gene signature. ROC, Receiver operating characteristic; AUC, Area under curve.

The KM plot and ROC curve were used to evaluate the performance of three-gene signature in predicting the outcome of the LADC patients. In training dataset, the OS between the low- and high-risk groups classified by our prognostic model was significantly different (*p* = 2.359E−05) (Fig. 4A). The area under curve (AUC) of ROC were 0.738 (Fig. 4B). Then, the performance of our model in LADC patients were further assessed with other common prognostic factors by univariate and multivariate Cox regression analysis. Although univariate Cox analysis indicated that tumor stage, T, N stage and our model all had prognostic effect (Fig. 4C), only three-gene signature can be used as independent prognostic factor (*p* < 0.001, Fig. 4D). In line with the results in the training dataset, the TME-related gene signature can well stratify the samples in three GEO testing datasets as low-risk and high-risk group (Figs. 5A–5C). The AUC of ROC curves of 3 and 5 years in the testing dataset were 0.646, 0.635 (GSE30219), 0.718, 0.569 (GSE3141) and 0.643, 0.65 (GSE50081) (Figs. 5D–5F). These results indicated that the three-gene prognostic model was robust in predicting the outcome of LADC patients.

### Function enrichment analysis of TME-related gene signature

**Figure 6:**
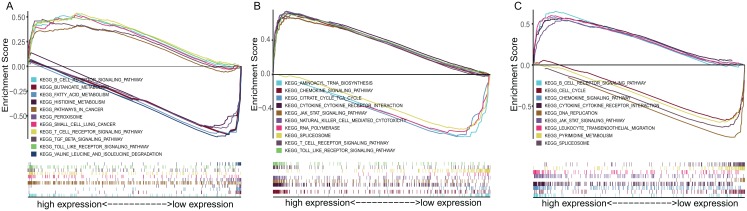
The GSEA enrichment analysis of TME-related gene signature. (A) ADAM12. (B) BTK. (C) ERG.

To explore the underlying mechanisms of the prognostic effects of three-gene signature, GSEA enrichment analysis was performed. The results suggested that highly expressed ADAM12 correlated with the activation of pathways such as SCLC, pathway in cancer, transforming growth factor beta signaling pathway, while lowly expressed ADAM12 associated with the metabolism pathway such as butanoate metabolism, fatty acid metabolism, histidine metabolism (Fig. 6A). Highly expressed BTK and ERG mainly correlated with the immune associated pathways such as cytokine–cytokine receptor interaction, JAK-STAT signaling pathway, while lowly expressed BTK and ERG associated with DNA replication, spliceosome pathway (Figs. 6B and 6C).

### Evaluation the immune status between low-risk and high-risk groups

**Figure 7:**
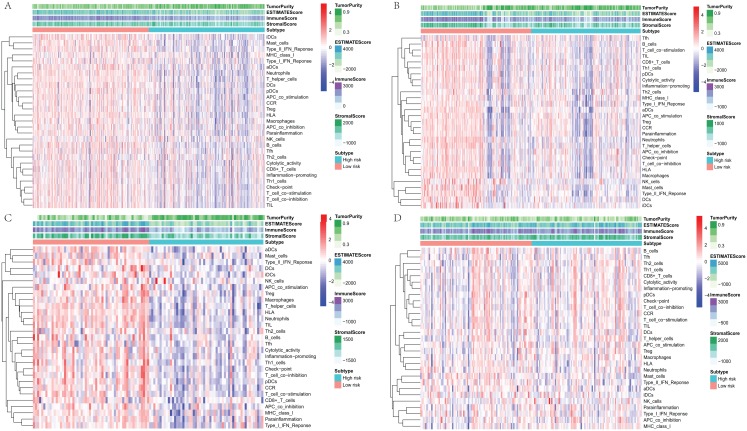
The heatmap of overall immune status and tumor purity of low- and high-risk groups analyzed by ssGSEA and ESTIMATE method. (A) The heatmap of TCGA dataset. (B) The heatmap of GSE30219. (C) The heatmap of GSE3141. (D) The heatmap of GSE50081. ssGSEA, single sample Gene Set Enrichment Analysis; ESTIMATE, Estimation of STromal and Immune cells in MAlignant Tumour tissues using Expression data.

**Figure 8:**
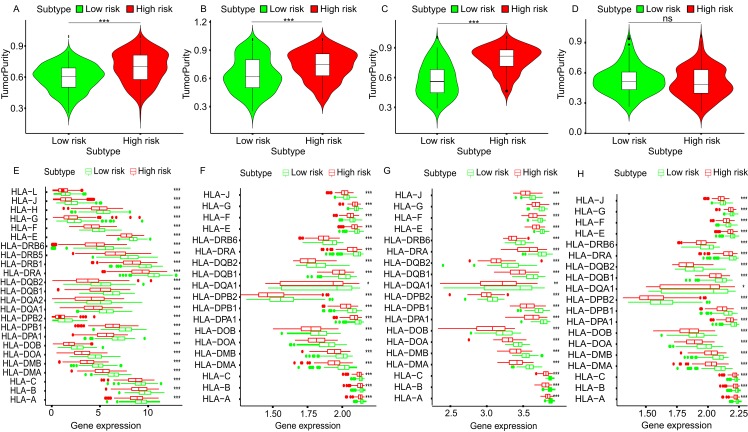
The tumor purity and HLA genes expression profiles of low- and high-risk groups in four datasets. The violin plots of tumor purity of low- and high-risk groups in TCGA dataset (A), GSE30219 (B), GSE3141 (C) and GSE50081 (D). The expression profiles of HLA genes of low- and high-risk groups in TCGA dataset (E), GSE30219 (F), GSE3141 (G) and GSE50081 (H). HLA, Human Leukocyte Antigen. ***p < 0.001, **p < 0.01. *p < 0.05. ns, *p* > 0.05.

To further explore the relationship between three-gene signature and immune system, ssGSEA method was used to assess the overall immune status of high-risk and low-risk groups by analyzing the expression profiles of the 29 immune signature genesets. The heatmap showed that in TCGA and three GEO datasets, the immune status of the low-risk and high-risk samples showed a certain degree of heterogeneity. Except GSE50081 (Fig.7D), the low-risk group in TCGA (Fig.7A), GSE30219 (Fig.7B) and GSE3141 (Fig.7C) showed more immune activities than that of high-risk group. Consistent with ssGSEA results, except GSE50081 (Fig. 8D), tumor purity of low-risk groups in TCGA (Fig.8A), GSE30219 (Fig.8B) and GSE3141 (Fig.8C) were significantly higher than that of high-risk groups, which suggested more infiltrated immune and stromal cells in the TME of low-risk samples. The HLA related genes play a key role in immune regulation. The analysis showed that the expression of key HLA genes in all four datasets was significantly higher in the low-risk group than in the high-risk group (Figs. 8E–8H). Interestingly, although there is no significant difference in the overall immune status and tumor purity between the high- and low-risk groups in the GSE50008 dataset, significant differences in the expression of HLA genes suggest that even if there is the relative same proportion of immune cells infiltration, the difference in the function of immune cells can also affect the prognosis of patients. However, the more specific mechanisms may need further research.

## Discussion

The TME plays an important role in the development of tumors. In order to identify prognostic biomarkers associated with LADC TME, we first identified the TME-related DEGs, and constructed a three-gene (ADAM12, BTK, ERG) signature by LASSO Cox regression model, and validated its association with OS in three GEO test datasets. The results indicated that the three-gene signature can well classified the LADC patients of training and testing datasets into high- and low-risk groups, and high-risk groups were associated with poorer prognosis. The univariate and multivariate Cox analysis confirmed the three-gene signature can be used as independent prognostic factor for predicting the patients’ outcome. The ROC analyses using training, testing datasets also proved the robusticity of our prognostic signature. Although the three genes all belong to stromal signature gene, the PPI results showed that there was a wide interaction between the stromal signature DEGs and the immune signature DEGs, suggesting that the two types of cells maybe extensively interact in LADC TME. Immune and stromal cells infiltrated in TME are composed of many different types of cells. On the one hand, as the most abundant stromal cell, the fibroblasts can form physical barriers to avoid the immune recognition and elimination of tumor cells, and they promote the tumor proliferation and metastasis by regulating the extracellular matrix and secreting related cytokines or growth factors (Chen & Song, 2019; Huang et al., 2010; Orimo et al., 2005; Scherz-Shouval et al., 2014; Seino et al., 2018; Zhang et al., 2013). On the other hand, some fibroblast subtypes also show anti-tumor activities (Brechbuhl et al., 2017; Özdemir et al., 2015; Rhim et al., 2014). The LADC patients with high stromal score have longer survival time. Whether it means these patients have more active anti-tumor stromal cells and what types of stromal cells play a major anti-tumor role in these patients’ TME remain to be further studied. Among the three signature genes, ADAM12 was overexpressed in SCLC and might serve as a potential prognostic biomarker of SCLC (Duan et al., 2019; Shao et al., 2014; Xiong et al., 2018). Current study reveals that p65BTK, a novel isoform of the BTK, is overexpressed in NSCLC, and may be a novel drug target (Giordano et al., 2019). Although ERG have no reported association with LADC, their functions in LADC initial and progress are worthy further study.

To study the potential molecular mechanism of prognostic effects of gene signature, GSEA analysis was conducted. The results show that the expression changes of genes in the prognostic model mainly affect the intercellular adhesion and the pathways related to immune regulation, which provides clues for the further research. To explore the state of immunity in TME, ssGSEA and ESTIMATE method were used to evaluate the overall immune status and tumor purity in LADC TME. Consistent with the GSEA pathway analysis, the overall immune activity of most low-risk groups was higher than that of the high-risk group. Correspondingly, the tumor purity was lower than that of the high-risk group, suggesting that more stromal cells and immune cells were infiltrated in the TME, and HLA expression analysis also showed that the key HLA genes in the low-risk group were highly expressed, suggesting that local immune regulation and response were more active, which partly explained the results of survival analysis. However, the GSE50081 dataset showed greater heterogeneity between the high- and low-risk groups, and the tumor purity was not statistically different, but the HLA gene expression trend was similar to other datasets, suggesting that although between high- and low-risk groups, there is no difference in the proportion of immune cells and stromal cells infiltrated at TME, but differences in immune cell function may also affect the prognosis of patients.

## Conclusions

We used LADC transcriptome data to identify TME-related DEGs. From the DEGs, a three-gene signature was constructed and validated for predicting the outcomes of LADC patients. Further study of these TME-related genes will provide a new understanding of the potential relationship between TME and LADC prognosis.

## Supplemental Information

10.7717/peerj.8128/supp-1 Supplemental Information 1 The differentially expressed TME-related genes.

10.7717/peerj.8128/supp-2 Supplemental Information 2 The GO enrichment result of TME-related DEGs.

10.7717/peerj.8128/supp-3 Supplemental Information 3 The KEGG analysis of TME-related DEGs.

## Additional Information and Declarations

### Data Availability

The following information was supplied regarding data availability: The expression profile and clinical data are all from the level 3 data available in the TCGA database (https://portal.gdc.cancer.gov/repository). Search terms: “Primary Site” IS “Bronchus and lung” AND “Project Id” IS “TCGA-LUAD” AND “Workflow Type” IS “HTSeq-FPKM” AND “Data Category” IS “TranscriptomeProfiling” AND “Data Type” IS “Gene Expression Quantification.” The microarray-basedexpression data of LADC patients and associated clinical information are available at Gene Expression Omnibus: GSE3141, GSE30219, GSE50081.
