# Supplementary material for: Identification of prognostic gene signature associated with microenvironment of lung adenocarcinoma
Source: PeerJ. 2019 Nov 29;7:e8128. doi: 10.7717/peerj.8128 (PMC6886493; doi:10.7717/peerj.8128)
Supplement: Supplemental Information 1 [file peerj-07-8128-s001.docx]

The differentially expressed TME-related genes.

| Gene | Log2(Fold Change) | pValue | FDR | Gene type |
| --- | --- | --- | --- | --- |
| LDB2 | -2.51107 | 3.76E-32 | 4.53E-30 | Stromal signature gene |
| LRRC15 | 2.419895 | 1.69E-11 | 3.45E-11 | Stromal signature gene |
| ITM2A | -1.9102 | 4.42E-28 | 5.33E-27 | Stromal signature gene |
| CDH5 | -2.45073 | 9.61E-32 | 5.37E-30 | Stromal signature gene |
| KCNJ8 | -1.07146 | 4.41E-22 | 2.08E-21 | Stromal signature gene |
| COMP | 3.05628 | 1.28E-15 | 3.36E-15 | Stromal signature gene |
| C1QB | -1.41515 | 5.59E-22 | 2.54E-21 | Stromal signature gene |
| THBS2 | 3.060913 | 2.53E-25 | 1.74E-24 | Stromal signature gene |
| COL10A1 | 4.687694 | 6.66E-30 | 1.34E-28 | Stromal signature gene |
| GMFG | -1.36309 | 1.81E-26 | 1.68E-25 | Immune signature gene |
| MS4A4A | -1.04237 | 7.14E-18 | 2.26E-17 | Stromal signature gene |
| COL6A3 | 1.427861 | 7.27E-12 | 1.54E-11 | Stromal signature gene |
| ADAM12 | 3.346858 | 7.62E-23 | 4.08E-22 | Stromal signature gene |
| ENPP2 | -1.20487 | 2.45E-09 | 4.19E-09 | Stromal signature gene |
| BTK | -1.24095 | 1.50E-23 | 8.84E-23 | Stromal signature gene |
| EVI2B | -1.09196 | 5.42E-20 | 2.07E-19 | Immune signature gene |
| MXRA5 | 1.840815 | 1.56E-12 | 3.45E-12 | Stromal signature gene |
| CD302 | -1.19091 | 6.86E-24 | 4.35E-23 | Immune signature gene |
| PDE2A | -1.6385 | 1.91E-24 | 1.28E-23 | Stromal signature gene |
| AOC3 | -2.52253 | 3.38E-32 | 4.53E-30 | Stromal signature gene |
| ECM2 | -1.09003 | 8.76E-18 | 2.74E-17 | Stromal signature gene |
| EMCN | -2.63481 | 4.09E-31 | 1.41E-29 | Stromal signature gene |
| PRF1 | -1.11818 | 8.06E-15 | 1.94E-14 | Immune signature gene |
| LST1 | -1.21807 | 1.76E-20 | 7.07E-20 | Immune signature gene |
| OLFML1 | -1.49466 | 7.71E-26 | 6.19E-25 | Stromal signature gene |
| LRRC32 | -2.30545 | 1.95E-28 | 2.66E-27 | Stromal signature gene |
| CILP | 2.645528 | 8.06E-11 | 1.57E-10 | Stromal signature gene |
| PCDH12 | -1.61928 | 6.86E-24 | 4.35E-23 | Stromal signature gene |
| FGR | -2.00425 | 4.32E-30 | 1.04E-28 | Immune signature gene |
| SIGLEC1 | -1.12573 | 1.02E-17 | 3.16E-17 | Stromal signature gene |
| ITGAL | -1.05988 | 6.20E-18 | 2.02E-17 | Immune signature gene |
| HCK | -1.16041 | 1.63E-21 | 7.03E-21 | Immune signature gene |
| FOXF1 | -2.68229 | 2.20E-31 | 8.82E-30 | Stromal signature gene |
| COL3A1 | 2.73118 | 5.98E-23 | 3.35E-22 | Stromal signature gene |
| ALOX5AP | -1.78996 | 1.08E-25 | 8.37E-25 | Immune signature gene |
| ADAM8 | 2.247355 | 5.03E-22 | 2.33E-21 | Immune signature gene |
| CD37 | -1.00339 | 1.58E-17 | 4.71E-17 | Immune signature gene |
| SERPING1 | -1.2734 | 1.79E-27 | 1.87E-26 | Stromal signature gene |
| VSIG4 | -2.23701 | 2.38E-28 | 3.02E-27 | Stromal signature gene |
| COL1A2 | 1.669728 | 5.05E-13 | 1.14E-12 | Stromal signature gene |
| DIO2 | 2.584132 | 1.54E-17 | 4.63E-17 | Stromal signature gene |
| ACTG2 | -1.18393 | 1.65E-15 | 4.22E-15 | Stromal signature gene |
| HLA-E | -1.2236 | 1.07E-27 | 1.23E-26 | Immune signature gene |
| CYBB | -1.14208 | 1.04E-15 | 2.75E-15 | Immune signature gene |
| PRKG1 | -1.20987 | 1.02E-22 | 5.25E-22 | Stromal signature gene |
| GREM1 | 5.127774 | 2.79E-26 | 2.49E-25 | Stromal signature gene |
| C1QA | -1.41443 | 7.94E-24 | 4.91E-23 | Stromal signature gene |
| PIK3R5 | -1.0701 | 2.21E-18 | 7.41E-18 | Stromal signature gene |
| ZEB2 | -1.13337 | 1.88E-25 | 1.34E-24 | Stromal signature gene |
| TENM4 | 1.963024 | 4.06E-17 | 1.19E-16 | Stromal signature gene |
| SELPLG | -1.32101 | 1.56E-26 | 1.51E-25 | Immune signature gene |
| CD27 | 1.407283 | 5.52E-11 | 1.09E-10 | Immune signature gene |
| CCDC69 | -1.37189 | 3.95E-29 | 6.80E-28 | Immune signature gene |
| P2RY14 | -1.29563 | 1.55E-20 | 6.34E-20 | Immune signature gene |
| TNFSF4 | 1.40205 | 1.40E-12 | 3.12E-12 | Stromal signature gene |
| BCL2A1 | -1.1094 | 1.56E-15 | 4.05E-15 | Immune signature gene |
| TYROBP | -1.37157 | 8.48E-23 | 4.45E-22 | Immune signature gene |
| WISP1 | 2.388091 | 2.94E-22 | 1.42E-21 | Stromal signature gene |
| RAMP3 | -3.14273 | 5.64E-32 | 4.53E-30 | Stromal signature gene |
| DCN | -1.33608 | 2.94E-21 | 1.24E-20 | Stromal signature gene |
| MMP3 | 4.041611 | 4.90E-17 | 1.42E-16 | Stromal signature gene |
| RASSF2 | -1.08486 | 8.21E-21 | 3.41E-20 | Immune signature gene |
| ARHGEF6 | -1.27294 | 4.87E-26 | 4.05E-25 | Immune signature gene |
| FCER1G | -1.09157 | 5.99E-20 | 2.26E-19 | Immune signature gene |
| MNDA | -1.29971 | 2.30E-22 | 1.13E-21 | Immune signature gene |
| NCF2 | -1.52957 | 4.18E-26 | 3.60E-25 | Immune signature gene |
| GIMAP4 | -1.34798 | 1.34E-25 | 1.01E-24 | Immune signature gene |
| CD93 | -2.07897 | 6.13E-30 | 1.34E-28 | Stromal signature gene |
| IL7R | -1.89239 | 1.84E-23 | 1.05E-22 | Immune signature gene |
| TCF21 | -2.98012 | 1.11E-31 | 5.37E-30 | Stromal signature gene |
| CXCL9 | 1.501963 | 0.000368 | 0.000475 | Stromal signature gene |
| MSR1 | -2.13989 | 1.99E-28 | 2.66E-27 | Stromal signature gene |
| COX7A1 | -1.84936 | 1.82E-29 | 3.37E-28 | Stromal signature gene |
| PTGIS | -1.15084 | 4.21E-18 | 1.39E-17 | Stromal signature gene |
| ERG | -2.0483 | 5.55E-31 | 1.65E-29 | Stromal signature gene |
| FPR1 | -1.76365 | 2.62E-19 | 9.44E-19 | Stromal signature gene |
| SAMHD1 | -1.00927 | 6.85E-23 | 3.75E-22 | Immune signature gene |
| ITIH5 | -1.9234 | 1.50E-27 | 1.64E-26 | Stromal signature gene |
| FAP | 2.418121 | 1.24E-23 | 7.48E-23 | Stromal signature gene |
| LAPTM5 | -1.0069 | 1.79E-19 | 6.52E-19 | Immune signature gene |
| GIMAP6 | -1.96027 | 6.16E-31 | 1.65E-29 | Immune signature gene |
| NCKAP1L | -1.0892 | 5.07E-20 | 1.97E-19 | Immune signature gene |
| CXCL14 | 4.386955 | 6.12E-11 | 1.20E-10 | Stromal signature gene |
| CD33 | -1.3092 | 1.27E-22 | 6.36E-22 | Stromal signature gene |
| IL18R1 | -1.38376 | 2.35E-14 | 5.50E-14 | Stromal signature gene |
| SULF1 | 2.670432 | 7.93E-22 | 3.48E-21 | Stromal signature gene |
| CXCL12 | -1.14392 | 9.86E-20 | 3.65E-19 | Stromal signature gene |
| CD52 | -2.24327 | 6.90E-29 | 1.11E-27 | Immune signature gene |
| FLI1 | -1.53936 | 1.56E-28 | 2.35E-27 | Immune signature gene |
| SFRP4 | 1.279449 | 1.02E-05 | 1.42E-05 | Stromal signature gene |
| LY86 | -1.27921 | 7.46E-22 | 3.33E-21 | Stromal signature gene |
| LMOD1 | -1.73891 | 1.39E-25 | 1.02E-24 | Stromal signature gene |
| PTGER4 | -1.4056 | 1.30E-26 | 1.31E-25 | Immune signature gene |

TME: Tumor Microenvironment. FDR: False Discovery Rate
