# Supplementary material for: Identification of prognostic gene signature associated with microenvironment of lung adenocarcinoma
Source: PeerJ. 2019 Nov 29;7:e8128. doi: 10.7717/peerj.8128 (PMC6886493; doi:10.7717/peerj.8128)
Supplement: Supplemental Information 2 [file peerj-07-8128-s002.docx]

The GO enrichment result of TME-related DEGs.

| ONTOLOGY | ID | Description | GeneRatio | BgRatio | qvalue | Count |
| --- | --- | --- | --- | --- | --- | --- |
| CC | GO:0062023 | collagen-containing extracellular matrix | 18/91 | 399/18678 | 6.65E-11 | 18 |
| CC | GO:0031012 | extracellular matrix | 19/91 | 468/18678 | 6.65E-11 | 19 |
| BP | GO:0002683 | negative regulation of immune system process | 17/86 | 451/17913 | 5.87E-08 | 17 |
| CC | GO:0005581 | collagen trimer | 8/91 | 86/18678 | 4.40E-07 | 8 |
| BP | GO:0030198 | extracellular matrix organization | 14/86 | 334/17913 | 4.59E-07 | 14 |
| BP | GO:0050727 | regulation of inflammatory response | 16/86 | 479/17913 | 4.59E-07 | 16 |
| BP | GO:0002694 | regulation of leukocyte activation | 16/86 | 492/17913 | 5.06E-07 | 16 |
| BP | GO:0043062 | extracellular structure organization | 14/86 | 387/17913 | 1.26E-06 | 14 |
| BP | GO:0032103 | positive regulation of response to external stimulus | 12/86 | 315/17913 | 8.44E-06 | 12 |
| BP | GO:0070664 | negative regulation of leukocyte proliferation | 7/86 | 71/17913 | 1.02E-05 | 7 |
| BP | GO:0070661 | leukocyte proliferation | 11/86 | 270/17913 | 1.25E-05 | 11 |
| BP | GO:0070663 | regulation of leukocyte proliferation | 10/86 | 214/17913 | 1.28E-05 | 10 |
| MF | GO:0005201 | extracellular matrix structural constituent | 9/84 | 158/16969 | 1.54E-05 | 9 |
| BP | GO:0002449 | lymphocyte mediated immunity | 11/86 | 293/17913 | 2.23E-05 | 11 |
| BP | GO:0002460 | adaptive immune response based on somatic recombination of immune receptors built from immunoglobulin superfamily domains | 11/86 | 295/17913 | 2.23E-05 | 11 |
| BP | GO:0002696 | positive regulation of leukocyte activation | 11/86 | 305/17913 | 2.81E-05 | 11 |
| BP | GO:0046651 | lymphocyte proliferation | 10/86 | 244/17913 | 2.81E-05 | 10 |
| BP | GO:0050866 | negative regulation of cell activation | 9/86 | 187/17913 | 2.81E-05 | 9 |
| BP | GO:0032943 | mononuclear cell proliferation | 10/86 | 246/17913 | 2.81E-05 | 10 |
| BP | GO:0050867 | positive regulation of cell activation | 11/86 | 319/17913 | 3.34E-05 | 11 |
| BP | GO:0050777 | negative regulation of immune response | 8/86 | 143/17913 | 3.34E-05 | 8 |
| BP | GO:1902107 | positive regulation of leukocyte differentiation | 8/86 | 143/17913 | 3.34E-05 | 8 |
| BP | GO:0051249 | regulation of lymphocyte activation | 12/86 | 401/17913 | 3.34E-05 | 12 |
| BP | GO:0007159 | leukocyte cell-cell adhesion | 11/86 | 328/17913 | 3.34E-05 | 11 |
| BP | GO:0050670 | regulation of lymphocyte proliferation | 9/86 | 199/17913 | 3.34E-05 | 9 |
| BP | GO:0032944 | regulation of mononuclear cell proliferation | 9/86 | 200/17913 | 3.34E-05 | 9 |
| BP | GO:0002521 | leukocyte differentiation | 13/86 | 485/17913 | 3.34E-05 | 13 |
| BP | GO:0032945 | negative regulation of mononuclear cell proliferation | 6/86 | 65/17913 | 4.04E-05 | 6 |
| BP | GO:0050672 | negative regulation of lymphocyte proliferation | 6/86 | 65/17913 | 4.04E-05 | 6 |
| BP | GO:0050729 | positive regulation of inflammatory response | 8/86 | 153/17913 | 4.09E-05 | 8 |
| BP | GO:0002673 | regulation of acute inflammatory response | 8/86 | 160/17913 | 5.53E-05 | 8 |
| BP | GO:0002526 | acute inflammatory response | 9/86 | 220/17913 | 5.84E-05 | 9 |
| BP | GO:0002695 | negative regulation of leukocyte activation | 8/86 | 163/17913 | 5.92E-05 | 8 |
| BP | GO:0002697 | regulation of immune effector process | 12/86 | 441/17913 | 6.26E-05 | 12 |
| BP | GO:0016064 | immunoglobulin mediated immune response | 8/86 | 171/17913 | 7.95E-05 | 8 |
| BP | GO:0019724 | B cell mediated immunity | 8/86 | 173/17913 | 8.40E-05 | 8 |
| BP | GO:0006909 | phagocytosis | 10/86 | 308/17913 | 9.86E-05 | 10 |
| BP | GO:1903708 | positive regulation of hemopoiesis | 8/86 | 182/17913 | 0.000115 | 8 |
| BP | GO:2000106 | regulation of leukocyte apoptotic process | 6/86 | 82/17913 | 0.000115 | 6 |
| BP | GO:0001501 | skeletal system development | 12/86 | 479/17913 | 0.00012 | 12 |
| BP | GO:0002703 | regulation of leukocyte mediated immunity | 8/86 | 185/17913 | 0.00012 | 8 |
| BP | GO:0051250 | negative regulation of lymphocyte activation | 7/86 | 132/17913 | 0.000129 | 7 |
| BP | GO:0043312 | neutrophil degranulation | 12/86 | 485/17913 | 0.000129 | 12 |
| BP | GO:0002283 | neutrophil activation involved in immune response | 12/86 | 488/17913 | 0.000134 | 12 |
| BP | GO:0002440 | production of molecular mediator of immune response | 8/86 | 191/17913 | 0.000137 | 8 |
| BP | GO:0002446 | neutrophil mediated immunity | 12/86 | 499/17913 | 0.000156 | 12 |
| BP | GO:0042119 | neutrophil activation | 12/86 | 499/17913 | 0.000156 | 12 |
| BP | GO:0002886 | regulation of myeloid leukocyte mediated immunity | 5/86 | 52/17913 | 0.000168 | 5 |
| BP | GO:1902105 | regulation of leukocyte differentiation | 9/86 | 267/17913 | 0.000176 | 9 |
| BP | GO:0006968 | cellular defense response | 5/86 | 53/17913 | 0.000176 | 5 |
| BP | GO:0045621 | positive regulation of lymphocyte differentiation | 6/86 | 93/17913 | 0.000178 | 6 |
| BP | GO:0007229 | integrin-mediated signaling pathway | 6/86 | 94/17913 | 0.000185 | 6 |
| BP | GO:0002377 | immunoglobulin production | 6/86 | 98/17913 | 0.000231 | 6 |
| BP | GO:0042110 | T cell activation | 11/86 | 443/17913 | 0.000258 | 11 |
| BP | GO:0042113 | B cell activation | 8/86 | 215/17913 | 0.000263 | 8 |
| BP | GO:0071887 | leukocyte apoptotic process | 6/86 | 103/17913 | 0.00029 | 6 |
| BP | GO:0002888 | positive regulation of myeloid leukocyte mediated immunity | 4/86 | 29/17913 | 0.000302 | 4 |
| BP | GO:1903037 | regulation of leukocyte cell-cell adhesion | 9/86 | 295/17913 | 0.000331 | 9 |
| CC | GO:0101003 | ficolin-1-rich granule membrane | 5/91 | 61/18678 | 0.000376 | 5 |
| CC | GO:0030667 | secretory granule membrane | 9/91 | 293/18678 | 0.000376 | 9 |
| CC | GO:0070820 | tertiary granule | 7/91 | 164/18678 | 0.000376 | 7 |
| BP | GO:0002675 | positive regulation of acute inflammatory response | 4/86 | 31/17913 | 0.000377 | 4 |
| BP | GO:0050900 | leukocyte migration | 11/86 | 467/17913 | 0.000377 | 11 |
| BP | GO:0050864 | regulation of B cell activation | 6/86 | 110/17913 | 0.000386 | 6 |
| BP | GO:0022407 | regulation of cell-cell adhesion | 10/86 | 389/17913 | 0.000436 | 10 |
| BP | GO:0031348 | negative regulation of defense response | 8/86 | 237/17913 | 0.000461 | 8 |
| BP | GO:0002698 | negative regulation of immune effector process | 6/86 | 116/17913 | 0.000497 | 6 |
| BP | GO:0045730 | respiratory burst | 4/86 | 34/17913 | 0.000504 | 4 |
| BP | GO:0002705 | positive regulation of leukocyte mediated immunity | 6/86 | 117/17913 | 0.000505 | 6 |
| BP | GO:0002863 | positive regulation of inflammatory response to antigenic stimulus | 3/86 | 12/17913 | 0.000529 | 3 |
| BP | GO:0045579 | positive regulation of B cell differentiation | 3/86 | 12/17913 | 0.000529 | 3 |
| BP | GO:0042100 | B cell proliferation | 5/86 | 71/17913 | 0.000529 | 5 |
| BP | GO:0007178 | transmembrane receptor protein serine/threonine kinase signaling pathway | 9/86 | 323/17913 | 0.000555 | 9 |
| BP | GO:0030098 | lymphocyte differentiation | 9/86 | 325/17913 | 0.000574 | 9 |
| CC | GO:0070821 | tertiary granule membrane | 5/91 | 73/18678 | 0.000594 | 5 |
| CC | GO:0101002 | ficolin-1-rich granule | 7/91 | 185/18678 | 0.000613 | 7 |
| BP | GO:0002714 | positive regulation of B cell mediated immunity | 4/86 | 37/17913 | 0.000627 | 4 |
| BP | GO:0002891 | positive regulation of immunoglobulin mediated immune response | 4/86 | 37/17913 | 0.000627 | 4 |
| BP | GO:0002864 | regulation of acute inflammatory response to antigenic stimulus | 3/86 | 13/17913 | 0.00063 | 3 |
| BP | GO:0007599 | hemostasis | 9/86 | 336/17913 | 0.000694 | 9 |
| BP | GO:0051251 | positive regulation of lymphocyte activation | 8/86 | 258/17913 | 0.000694 | 8 |
| BP | GO:0060348 | bone development | 7/86 | 189/17913 | 0.000709 | 7 |
| BP | GO:0030889 | negative regulation of B cell proliferation | 3/86 | 14/17913 | 0.000756 | 3 |
| BP | GO:1903557 | positive regulation of tumor necrosis factor superfamily cytokine production | 5/86 | 81/17913 | 0.000869 | 5 |
| BP | GO:0002699 | positive regulation of immune effector process | 7/86 | 198/17913 | 0.000905 | 7 |
| BP | GO:0002822 | regulation of adaptive immune response based on somatic recombination of immune receptors built from immunoglobulin superfamily domains | 6/86 | 135/17913 | 0.000905 | 6 |
| BP | GO:0045088 | regulation of innate immune response | 10/86 | 440/17913 | 0.000905 | 10 |
| BP | GO:0002706 | regulation of lymphocyte mediated immunity | 6/86 | 136/17913 | 0.000905 | 6 |
| BP | GO:0002718 | regulation of cytokine production involved in immune response | 5/86 | 83/17913 | 0.000905 | 5 |
| BP | GO:0045582 | positive regulation of T cell differentiation | 5/86 | 83/17913 | 0.000905 | 5 |
| BP | GO:0043303 | mast cell degranulation | 4/86 | 43/17913 | 0.000965 | 4 |
| BP | GO:0001819 | positive regulation of cytokine production | 10/86 | 446/17913 | 0.000973 | 10 |
| BP | GO:0002279 | mast cell activation involved in immune response | 4/86 | 45/17913 | 0.001103 | 4 |
| BP | GO:0002448 | mast cell mediated immunity | 4/86 | 45/17913 | 0.001103 | 4 |
| BP | GO:0030199 | collagen fibril organization | 4/86 | 45/17913 | 0.001103 | 4 |
| BP | GO:0002456 | T cell mediated immunity | 5/86 | 88/17913 | 0.001116 | 5 |
| BP | GO:0002824 | positive regulation of adaptive immune response based on somatic recombination of immune receptors built from immunoglobulin superfamily domains | 5/86 | 89/17913 | 0.001163 | 5 |
| BP | GO:2000107 | negative regulation of leukocyte apoptotic process | 4/86 | 46/17913 | 0.001163 | 4 |
| BP | GO:0001818 | negative regulation of cytokine production | 8/86 | 290/17913 | 0.001252 | 8 |
| BP | GO:0002708 | positive regulation of lymphocyte mediated immunity | 5/86 | 91/17913 | 0.001252 | 5 |
| CC | GO:0035579 | specific granule membrane | 5/91 | 91/18678 | 0.001335 | 5 |
| BP | GO:0002819 | regulation of adaptive immune response | 6/86 | 150/17913 | 0.001372 | 6 |
| BP | GO:0002821 | positive regulation of adaptive immune response | 5/86 | 94/17913 | 0.00143 | 5 |
| BP | GO:0030168 | platelet activation | 6/86 | 152/17913 | 0.00144 | 6 |
| BP | GO:0045765 | regulation of angiogenesis | 9/86 | 383/17913 | 0.00144 | 9 |
| BP | GO:1900047 | negative regulation of hemostasis | 4/86 | 50/17913 | 0.001499 | 4 |
| BP | GO:1903555 | regulation of tumor necrosis factor superfamily cytokine production | 6/86 | 155/17913 | 0.001556 | 6 |
| BP | GO:0002712 | regulation of B cell mediated immunity | 4/86 | 51/17913 | 0.001556 | 4 |
| BP | GO:0002889 | regulation of immunoglobulin mediated immune response | 4/86 | 51/17913 | 0.001556 | 4 |
| BP | GO:0070228 | regulation of lymphocyte apoptotic process | 4/86 | 51/17913 | 0.001556 | 4 |
| BP | GO:0002720 | positive regulation of cytokine production involved in immune response | 4/86 | 52/17913 | 0.001657 | 4 |
| BP | GO:0050863 | regulation of T cell activation | 8/86 | 307/17913 | 0.001657 | 8 |
| BP | GO:0002438 | acute inflammatory response to antigenic stimulus | 3/86 | 20/17913 | 0.001665 | 3 |
| BP | GO:0071706 | tumor necrosis factor superfamily cytokine production | 6/86 | 161/17913 | 0.00178 | 6 |
| BP | GO:0002367 | cytokine production involved in immune response | 5/86 | 101/17913 | 0.00178 | 5 |
| BP | GO:0030217 | T cell differentiation | 7/86 | 234/17913 | 0.001871 | 7 |
| BP | GO:0045619 | regulation of lymphocyte differentiation | 6/86 | 164/17913 | 0.001927 | 6 |
| BP | GO:0071559 | response to transforming growth factor beta | 7/86 | 239/17913 | 0.002093 | 7 |
| BP | GO:0030888 | regulation of B cell proliferation | 4/86 | 57/17913 | 0.002168 | 4 |
| BP | GO:0045576 | mast cell activation | 4/86 | 57/17913 | 0.002168 | 4 |
| BP | GO:0045824 | negative regulation of innate immune response | 4/86 | 57/17913 | 0.002168 | 4 |
| BP | GO:0032753 | positive regulation of interleukin-4 production | 3/86 | 23/17913 | 0.002354 | 3 |
| BP | GO:0045123 | cellular extravasation | 4/86 | 59/17913 | 0.002436 | 4 |
| BP | GO:0007596 | blood coagulation | 8/86 | 331/17913 | 0.002483 | 8 |
| BP | GO:1901342 | regulation of vasculature development | 9/86 | 425/17913 | 0.002589 | 9 |
| BP | GO:0002861 | regulation of inflammatory response to antigenic stimulus | 3/86 | 24/17913 | 0.002589 | 3 |
| BP | GO:0002709 | regulation of T cell mediated immunity | 4/86 | 61/17913 | 0.002678 | 4 |
| BP | GO:0050817 | coagulation | 8/86 | 337/17913 | 0.002711 | 8 |
| BP | GO:0032102 | negative regulation of response to external stimulus | 8/86 | 343/17913 | 0.003029 | 8 |
| BP | GO:0002691 | regulation of cellular extravasation | 3/86 | 26/17913 | 0.003192 | 3 |
| BP | GO:0002685 | regulation of leukocyte migration | 6/86 | 186/17913 | 0.003371 | 6 |
| BP | GO:0032673 | regulation of interleukin-4 production | 3/86 | 27/17913 | 0.003492 | 3 |
| BP | GO:0043304 | regulation of mast cell degranulation | 3/86 | 27/17913 | 0.003492 | 3 |
| BP | GO:0032418 | lysosome localization | 4/86 | 67/17913 | 0.003602 | 4 |
| BP | GO:0070227 | lymphocyte apoptotic process | 4/86 | 67/17913 | 0.003602 | 4 |
| BP | GO:0061515 | myeloid cell development | 4/86 | 68/17913 | 0.003745 | 4 |
| BP | GO:0045577 | regulation of B cell differentiation | 3/86 | 28/17913 | 0.003745 | 3 |
| BP | GO:0050869 | negative regulation of B cell activation | 3/86 | 28/17913 | 0.003745 | 3 |
| BP | GO:0050871 | positive regulation of B cell activation | 4/86 | 70/17913 | 0.004099 | 4 |
| BP | GO:0033006 | regulation of mast cell activation involved in immune response | 3/86 | 29/17913 | 0.004099 | 3 |
| BP | GO:0050870 | positive regulation of T cell activation | 6/86 | 198/17913 | 0.004351 | 6 |
| MF | GO:0030020 | extracellular matrix structural constituent conferring tensile strength | 4/84 | 41/16969 | 0.004455 | 4 |
| BP | GO:1903706 | regulation of hemopoiesis | 9/86 | 468/17913 | 0.004603 | 9 |
| BP | GO:0043367 | CD4-positive, alpha-beta T cell differentiation | 4/86 | 74/17913 | 0.004954 | 4 |
| BP | GO:0046631 | alpha-beta T cell activation | 5/86 | 133/17913 | 0.005019 | 5 |
| BP | GO:1900046 | regulation of hemostasis | 4/86 | 75/17913 | 0.005137 | 4 |
| BP | GO:0032633 | interleukin-4 production | 3/86 | 32/17913 | 0.005225 | 3 |
| BP | GO:0070232 | regulation of T cell apoptotic process | 3/86 | 32/17913 | 0.005225 | 3 |
| BP | GO:1903532 | positive regulation of secretion by cell | 8/86 | 381/17913 | 0.005268 | 8 |
| BP | GO:0001913 | T cell mediated cytotoxicity | 3/86 | 33/17913 | 0.005604 | 3 |
| BP | GO:0042554 | superoxide anion generation | 3/86 | 33/17913 | 0.005604 | 3 |
| BP | GO:0002700 | regulation of production of molecular mediator of immune response | 5/86 | 138/17913 | 0.005646 | 5 |
| BP | GO:0032760 | positive regulation of tumor necrosis factor production | 4/86 | 78/17913 | 0.005669 | 4 |
| BP | GO:0045785 | positive regulation of cell adhesion | 8/86 | 388/17913 | 0.005713 | 8 |
| BP | GO:0045580 | regulation of T cell differentiation | 5/86 | 139/17913 | 0.005713 | 5 |
| BP | GO:2000403 | positive regulation of lymphocyte migration | 3/86 | 34/17913 | 0.005889 | 3 |
| BP | GO:1903039 | positive regulation of leukocyte cell-cell adhesion | 6/86 | 214/17913 | 0.005889 | 6 |
| BP | GO:0050878 | regulation of body fluid levels | 9/86 | 499/17913 | 0.006558 | 9 |
| BP | GO:0031341 | regulation of cell killing | 4/86 | 82/17913 | 0.006573 | 4 |
| BP | GO:0042129 | regulation of T cell proliferation | 5/86 | 149/17913 | 0.007565 | 5 |
| BP | GO:0051047 | positive regulation of secretion | 8/86 | 409/17913 | 0.007709 | 8 |
| BP | GO:0002711 | positive regulation of T cell mediated immunity | 3/86 | 38/17913 | 0.007875 | 3 |
| BP | GO:0032680 | regulation of tumor necrosis factor production | 5/86 | 151/17913 | 0.007875 | 5 |
| BP | GO:0002286 | T cell activation involved in immune response | 4/86 | 88/17913 | 0.008294 | 4 |
| BP | GO:0033003 | regulation of mast cell activation | 3/86 | 39/17913 | 0.008359 | 3 |
| BP | GO:0071560 | cellular response to transforming growth factor beta stimulus | 6/86 | 233/17913 | 0.00863 | 6 |
| BP | GO:0032640 | tumor necrosis factor production | 5/86 | 155/17913 | 0.00863 | 5 |
| BP | GO:0009615 | response to virus | 7/86 | 323/17913 | 0.008737 | 7 |
| BP | GO:0035710 | CD4-positive, alpha-beta T cell activation | 4/86 | 90/17913 | 0.008737 | 4 |
| BP | GO:0002285 | lymphocyte activation involved in immune response | 5/86 | 158/17913 | 0.009226 | 5 |
| BP | GO:0006911 | phagocytosis, engulfment | 3/86 | 41/17913 | 0.009276 | 3 |
| BP | GO:0098883 | synapse pruning | 2/86 | 10/17913 | 0.009276 | 2 |
| BP | GO:0002702 | positive regulation of production of molecular mediator of immune response | 4/86 | 93/17913 | 0.009572 | 4 |
| BP | GO:0048010 | vascular endothelial growth factor receptor signaling pathway | 4/86 | 93/17913 | 0.009572 | 4 |
| BP | GO:0051090 | regulation of DNA-binding transcription factor activity | 8/86 | 429/17913 | 0.009592 | 8 |
| BP | GO:0030336 | negative regulation of cell migration | 7/86 | 332/17913 | 0.009802 | 7 |
| BP | GO:0007188 | adenylate cyclase-modulating G protein-coupled receptor signaling pathway | 5/86 | 162/17913 | 0.009881 | 5 |
| BP | GO:0043300 | regulation of leukocyte degranulation | 3/86 | 43/17913 | 0.010266 | 3 |
| BP | GO:0042116 | macrophage activation | 4/86 | 96/17913 | 0.010452 | 4 |
| BP | GO:0033089 | positive regulation of T cell differentiation in thymus | 2/86 | 11/17913 | 0.01066 | 2 |
| BP | GO:0051024 | positive regulation of immunoglobulin secretion | 2/86 | 11/17913 | 0.01066 | 2 |
| BP | GO:0001909 | leukocyte mediated cytotoxicity | 4/86 | 97/17913 | 0.01066 | 4 |
| BP | GO:0002437 | inflammatory response to antigenic stimulus | 3/86 | 44/17913 | 0.01066 | 3 |
| BP | GO:0022409 | positive regulation of cell-cell adhesion | 6/86 | 251/17913 | 0.011311 | 6 |
| BP | GO:0046632 | alpha-beta T cell differentiation | 4/86 | 99/17913 | 0.011311 | 4 |
| BP | GO:0022408 | negative regulation of cell-cell adhesion | 5/86 | 169/17913 | 0.011311 | 5 |
| BP | GO:0097529 | myeloid leukocyte migration | 5/86 | 170/17913 | 0.011542 | 5 |
| BP | GO:0060840 | artery development | 4/86 | 100/17913 | 0.011542 | 4 |
| BP | GO:2000146 | negative regulation of cell motility | 7/86 | 347/17913 | 0.01166 | 7 |
| BP | GO:0070231 | T cell apoptotic process | 3/86 | 46/17913 | 0.01166 | 3 |
| BP | GO:0050714 | positive regulation of protein secretion | 6/86 | 256/17913 | 0.012072 | 6 |
| BP | GO:0070613 | regulation of protein processing | 5/86 | 173/17913 | 0.012077 | 5 |
| BP | GO:0031623 | receptor internalization | 4/86 | 102/17913 | 0.012077 | 4 |
| BP | GO:1903317 | regulation of protein maturation | 5/86 | 175/17913 | 0.012619 | 5 |
| BP | GO:0002686 | negative regulation of leukocyte migration | 3/86 | 48/17913 | 0.012697 | 3 |
| BP | GO:0002704 | negative regulation of leukocyte mediated immunity | 3/86 | 48/17913 | 0.012697 | 3 |
| BP | GO:0032653 | regulation of interleukin-10 production | 3/86 | 48/17913 | 0.012697 | 3 |
| BP | GO:0042098 | T cell proliferation | 5/86 | 177/17913 | 0.012983 | 5 |
| BP | GO:0002688 | regulation of leukocyte chemotaxis | 4/86 | 105/17913 | 0.013009 | 4 |
| BP | GO:0007187 | G protein-coupled receptor signaling pathway, coupled to cyclic nucleotide second messenger | 5/86 | 178/17913 | 0.013167 | 5 |
| BP | GO:0030195 | negative regulation of blood coagulation | 3/86 | 49/17913 | 0.013195 | 3 |
| BP | GO:0002921 | negative regulation of humoral immune response | 2/86 | 13/17913 | 0.013288 | 2 |
| BP | GO:0033008 | positive regulation of mast cell activation involved in immune response | 2/86 | 13/17913 | 0.013288 | 2 |
| BP | GO:0043306 | positive regulation of mast cell degranulation | 2/86 | 13/17913 | 0.013288 | 2 |
| BP | GO:0031343 | positive regulation of cell killing | 3/86 | 50/17913 | 0.01363 | 3 |
| BP | GO:0099024 | plasma membrane invagination | 3/86 | 50/17913 | 0.01363 | 3 |
| BP | GO:0090130 | tissue migration | 7/86 | 363/17913 | 0.01376 | 7 |
| BP | GO:0030282 | bone mineralization | 4/86 | 108/17913 | 0.013767 | 4 |
| BP | GO:0051271 | negative regulation of cellular component movement | 7/86 | 365/17913 | 0.014051 | 7 |
| BP | GO:0032613 | interleukin-10 production | 3/86 | 51/17913 | 0.014142 | 3 |
| MF | GO:0005178 | integrin binding | 5/84 | 113/16969 | 0.014687 | 5 |
| BP | GO:0072677 | eosinophil migration | 2/86 | 14/17913 | 0.014914 | 2 |
| CC | GO:0042581 | specific granule | 5/91 | 160/18678 | 0.0153 | 5 |
| CC | GO:0005583 | fibrillar collagen trimer | 2/91 | 11/18678 | 0.0153 | 2 |
| CC | GO:0098643 | banded collagen fibril | 2/91 | 11/18678 | 0.0153 | 2 |
| BP | GO:0002793 | positive regulation of peptide secretion | 6/86 | 275/17913 | 0.015336 | 6 |
| BP | GO:0000768 | syncytium formation by plasma membrane fusion | 3/86 | 53/17913 | 0.015336 | 3 |
| BP | GO:0050819 | negative regulation of coagulation | 3/86 | 53/17913 | 0.015336 | 3 |
| BP | GO:0050853 | B cell receptor signaling pathway | 3/86 | 53/17913 | 0.015336 | 3 |
| BP | GO:0140253 | cell-cell fusion | 3/86 | 53/17913 | 0.015336 | 3 |
| BP | GO:0030595 | leukocyte chemotaxis | 5/86 | 188/17913 | 0.015343 | 5 |
| BP | GO:0030183 | B cell differentiation | 4/86 | 113/17913 | 0.015459 | 4 |
| BP | GO:1901222 | regulation of NIK/NF-kappaB signaling | 4/86 | 114/17913 | 0.015886 | 4 |
| BP | GO:0040013 | negative regulation of locomotion | 7/86 | 377/17913 | 0.015973 | 7 |
| BP | GO:0032736 | positive regulation of interleukin-13 production | 2/86 | 15/17913 | 0.016108 | 2 |
| BP | GO:0042535 | positive regulation of tumor necrosis factor biosynthetic process | 2/86 | 15/17913 | 0.016108 | 2 |
| BP | GO:1900119 | positive regulation of execution phase of apoptosis | 2/86 | 15/17913 | 0.016108 | 2 |
| BP | GO:2001185 | regulation of CD8-positive, alpha-beta T cell activation | 2/86 | 15/17913 | 0.016108 | 2 |
| BP | GO:0006949 | syncytium formation | 3/86 | 55/17913 | 0.01634 | 3 |
| BP | GO:0030449 | regulation of complement activation | 4/86 | 116/17913 | 0.016372 | 4 |
| BP | GO:2000257 | regulation of protein activation cascade | 4/86 | 117/17913 | 0.016813 | 4 |
| CC | GO:0098797 | plasma membrane protein complex | 8/91 | 451/18678 | 0.018054 | 8 |
| BP | GO:0010324 | membrane invagination | 3/86 | 58/17913 | 0.01854 | 3 |
| BP | GO:0042093 | T-helper cell differentiation | 3/86 | 58/17913 | 0.01854 | 3 |
| BP | GO:0002687 | positive regulation of leukocyte migration | 4/86 | 121/17913 | 0.01854 | 4 |
| BP | GO:1903038 | negative regulation of leukocyte cell-cell adhesion | 4/86 | 121/17913 | 0.01854 | 4 |
| BP | GO:0007254 | JNK cascade | 5/86 | 200/17913 | 0.01854 | 5 |
| BP | GO:0009612 | response to mechanical stimulus | 5/86 | 200/17913 | 0.01854 | 5 |
| BP | GO:0072001 | renal system development | 6/86 | 291/17913 | 0.01854 | 6 |
| BP | GO:0045766 | positive regulation of angiogenesis | 5/86 | 203/17913 | 0.019484 | 5 |
| BP | GO:0070233 | negative regulation of T cell apoptotic process | 2/86 | 17/17913 | 0.019484 | 2 |
| BP | GO:0016485 | protein processing | 6/86 | 295/17913 | 0.019484 | 6 |
| BP | GO:0032496 | response to lipopolysaccharide | 6/86 | 295/17913 | 0.019484 | 6 |
| MF | GO:0008009 | chemokine activity | 3/84 | 30/16969 | 0.019525 | 3 |
| BP | GO:0002294 | CD4-positive, alpha-beta T cell differentiation involved in immune response | 3/86 | 60/17913 | 0.019577 | 3 |
| BP | GO:2000401 | regulation of lymphocyte migration | 3/86 | 60/17913 | 0.019577 | 3 |
| BP | GO:0032956 | regulation of actin cytoskeleton organization | 6/86 | 297/17913 | 0.019882 | 6 |
| BP | GO:0002287 | alpha-beta T cell activation involved in immune response | 3/86 | 61/17913 | 0.020166 | 3 |
| BP | GO:0002293 | alpha-beta T cell differentiation involved in immune response | 3/86 | 61/17913 | 0.020166 | 3 |
| BP | GO:0046635 | positive regulation of alpha-beta T cell activation | 3/86 | 61/17913 | 0.020166 | 3 |
| BP | GO:0001911 | negative regulation of leukocyte mediated cytotoxicity | 2/86 | 18/17913 | 0.02081 | 2 |
| BP | GO:0033005 | positive regulation of mast cell activation | 2/86 | 18/17913 | 0.02081 | 2 |
| BP | GO:0046641 | positive regulation of alpha-beta T cell proliferation | 2/86 | 18/17913 | 0.02081 | 2 |
| BP | GO:0048557 | embryonic digestive tract morphogenesis | 2/86 | 18/17913 | 0.02081 | 2 |
| BP | GO:0051023 | regulation of immunoglobulin secretion | 2/86 | 18/17913 | 0.02081 | 2 |
| BP | GO:0006956 | complement activation | 4/86 | 128/17913 | 0.021011 | 4 |
| BP | GO:0030101 | natural killer cell activation | 3/86 | 63/17913 | 0.021454 | 3 |
| CC | GO:0005767 | secondary lysosome | 2/91 | 14/18678 | 0.021493 | 2 |
| MF | GO:0005126 | cytokine receptor binding | 6/84 | 210/16969 | 0.022068 | 6 |
| BP | GO:0001910 | regulation of leukocyte mediated cytotoxicity | 3/86 | 64/17913 | 0.022247 | 3 |
| BP | GO:0071230 | cellular response to amino acid stimulus | 3/86 | 64/17913 | 0.022247 | 3 |
| BP | GO:0002726 | positive regulation of T cell cytokine production | 2/86 | 19/17913 | 0.022253 | 2 |
| BP | GO:0032700 | negative regulation of interleukin-17 production | 2/86 | 19/17913 | 0.022253 | 2 |
| BP | GO:0045063 | T-helper 1 cell differentiation | 2/86 | 19/17913 | 0.022253 | 2 |
| BP | GO:0090280 | positive regulation of calcium ion import | 2/86 | 19/17913 | 0.022253 | 2 |
| BP | GO:2000010 | positive regulation of protein localization to cell surface | 2/86 | 19/17913 | 0.022253 | 2 |
| BP | GO:0007160 | cell-matrix adhesion | 5/86 | 214/17913 | 0.022253 | 5 |
| BP | GO:0002237 | response to molecule of bacterial origin | 6/86 | 309/17913 | 0.022301 | 6 |
| BP | GO:0006801 | superoxide metabolic process | 3/86 | 65/17913 | 0.02241 | 3 |
| BP | GO:0030858 | positive regulation of epithelial cell differentiation | 3/86 | 65/17913 | 0.02241 | 3 |
| MF | GO:0005125 | cytokine activity | 5/84 | 154/16969 | 0.022584 | 5 |
| MF | GO:0045236 | CXCR chemokine receptor binding | 2/84 | 10/16969 | 0.022584 | 2 |
| MF | GO:0005172 | vascular endothelial growth factor receptor binding | 2/84 | 11/16969 | 0.022584 | 2 |
| MF | GO:0036122 | BMP binding | 2/84 | 11/16969 | 0.022584 | 2 |
| MF | GO:0048407 | platelet-derived growth factor binding | 2/84 | 11/16969 | 0.022584 | 2 |
| MF | GO:0019955 | cytokine binding | 4/84 | 100/16969 | 0.022584 | 4 |
| MF | GO:0030553 | cGMP binding | 2/84 | 12/16969 | 0.022584 | 2 |
| MF | GO:0042379 | chemokine receptor binding | 3/84 | 47/16969 | 0.022584 | 3 |
| BP | GO:0002292 | T cell differentiation involved in immune response | 3/86 | 66/17913 | 0.023303 | 3 |
| BP | GO:0030509 | BMP signaling pathway | 4/86 | 134/17913 | 0.023378 | 4 |
| BP | GO:0002920 | regulation of humoral immune response | 4/86 | 135/17913 | 0.023911 | 4 |
| BP | GO:0032816 | positive regulation of natural killer cell activation | 2/86 | 20/17913 | 0.02397 | 2 |
| BP | GO:1903010 | regulation of bone development | 2/86 | 21/17913 | 0.026212 | 2 |
| BP | GO:0032675 | regulation of interleukin-6 production | 4/86 | 139/17913 | 0.026212 | 4 |
| BP | GO:0031589 | cell-substrate adhesion | 6/86 | 322/17913 | 0.026289 | 6 |
| BP | GO:0001655 | urogenital system development | 6/86 | 324/17913 | 0.026982 | 6 |
| BP | GO:0110053 | regulation of actin filament organization | 5/86 | 228/17913 | 0.027742 | 5 |
| BP | GO:0031342 | negative regulation of cell killing | 2/86 | 22/17913 | 0.027764 | 2 |
| BP | GO:0032656 | regulation of interleukin-13 production | 2/86 | 22/17913 | 0.027764 | 2 |
| BP | GO:0033622 | integrin activation | 2/86 | 22/17913 | 0.027764 | 2 |
| BP | GO:0036037 | CD8-positive, alpha-beta T cell activation | 2/86 | 22/17913 | 0.027764 | 2 |
| BP | GO:0043302 | positive regulation of leukocyte degranulation | 2/86 | 22/17913 | 0.027764 | 2 |
| BP | GO:0048305 | immunoglobulin secretion | 2/86 | 22/17913 | 0.027764 | 2 |
| BP | GO:1904018 | positive regulation of vasculature development | 5/86 | 232/17913 | 0.029057 | 5 |
| BP | GO:0061045 | negative regulation of wound healing | 3/86 | 73/17913 | 0.029074 | 3 |
| BP | GO:0090066 | regulation of anatomical structure size | 7/86 | 442/17913 | 0.029703 | 7 |
| BP | GO:0060143 | positive regulation of syncytium formation by plasma membrane fusion | 2/86 | 23/17913 | 0.029865 | 2 |
| BP | GO:0030193 | regulation of blood coagulation | 3/86 | 74/17913 | 0.029865 | 3 |
| BP | GO:0008064 | regulation of actin polymerization or depolymerization | 4/86 | 147/17913 | 0.02995 | 4 |
| BP | GO:0071772 | response to BMP | 4/86 | 147/17913 | 0.02995 | 4 |
| BP | GO:0071773 | cellular response to BMP stimulus | 4/86 | 147/17913 | 0.02995 | 4 |
| BP | GO:0002479 | antigen processing and presentation of exogenous peptide antigen via MHC class I, TAP-dependent | 3/86 | 75/17913 | 0.030338 | 3 |
| BP | GO:1901224 | positive regulation of NIK/NF-kappaB signaling | 3/86 | 75/17913 | 0.030338 | 3 |
| BP | GO:0030832 | regulation of actin filament length | 4/86 | 148/17913 | 0.030338 | 4 |
| BP | GO:0032635 | interleukin-6 production | 4/86 | 149/17913 | 0.030769 | 4 |
| BP | GO:0051092 | positive regulation of NF-kappaB transcription factor activity | 4/86 | 149/17913 | 0.030769 | 4 |
| BP | GO:0048844 | artery morphogenesis | 3/86 | 76/17913 | 0.030769 | 3 |
| BP | GO:0150076 | neuroinflammatory response | 3/86 | 76/17913 | 0.030769 | 3 |
| BP | GO:0001914 | regulation of T cell mediated cytotoxicity | 2/86 | 24/17913 | 0.030769 | 2 |
| BP | GO:0002092 | positive regulation of receptor internalization | 2/86 | 24/17913 | 0.030769 | 2 |
| BP | GO:0031954 | positive regulation of protein autophosphorylation | 2/86 | 24/17913 | 0.030769 | 2 |
| BP | GO:0033081 | regulation of T cell differentiation in thymus | 2/86 | 24/17913 | 0.030769 | 2 |
| BP | GO:0032970 | regulation of actin filament-based process | 6/86 | 341/17913 | 0.031214 | 6 |
| BP | GO:0072376 | protein activation cascade | 4/86 | 151/17913 | 0.031413 | 4 |
| MF | GO:0001846 | opsonin binding | 2/84 | 15/16969 | 0.031532 | 2 |
| BP | GO:0038093 | Fc receptor signaling pathway | 5/86 | 241/17913 | 0.031565 | 5 |
| BP | GO:0006936 | muscle contraction | 6/86 | 343/17913 | 0.031761 | 6 |
| BP | GO:0022617 | extracellular matrix disassembly | 3/86 | 78/17913 | 0.032102 | 3 |
| BP | GO:0042590 | antigen processing and presentation of exogenous peptide antigen via MHC class I | 3/86 | 78/17913 | 0.032102 | 3 |
| BP | GO:0050708 | regulation of protein secretion | 7/86 | 456/17913 | 0.032102 | 7 |
| BP | GO:0032616 | interleukin-13 production | 2/86 | 25/17913 | 0.032102 | 2 |
| BP | GO:0042730 | fibrinolysis | 2/86 | 25/17913 | 0.032102 | 2 |
| BP | GO:0050901 | leukocyte tethering or rolling | 2/86 | 25/17913 | 0.032102 | 2 |
| BP | GO:0090025 | regulation of monocyte chemotaxis | 2/86 | 25/17913 | 0.032102 | 2 |
| CC | GO:0098644 | complex of collagen trimers | 2/91 | 18/18678 | 0.032479 | 2 |
| CC | GO:0045335 | phagocytic vesicle | 4/91 | 128/18678 | 0.032479 | 4 |
| BP | GO:0031214 | biomineral tissue development | 4/86 | 154/17913 | 0.032513 | 4 |
| BP | GO:0050818 | regulation of coagulation | 3/86 | 79/17913 | 0.032567 | 3 |
| MF | GO:0050664 | oxidoreductase activity, acting on NAD(P)H, oxygen as acceptor | 2/84 | 16/16969 | 0.033527 | 2 |
| BP | GO:0001906 | cell killing | 4/86 | 156/17913 | 0.033655 | 4 |
| BP | GO:0002825 | regulation of T-helper 1 type immune response | 2/86 | 26/17913 | 0.033655 | 2 |
| BP | GO:0019934 | cGMP-mediated signaling | 2/86 | 26/17913 | 0.033655 | 2 |
| BP | GO:0060142 | regulation of syncytium formation by plasma membrane fusion | 2/86 | 26/17913 | 0.033655 | 2 |
| BP | GO:0072202 | cell differentiation involved in metanephros development | 2/86 | 26/17913 | 0.033655 | 2 |
| BP | GO:0002690 | positive regulation of leukocyte chemotaxis | 3/86 | 81/17913 | 0.033655 | 3 |
| BP | GO:0014068 | positive regulation of phosphatidylinositol 3-kinase signaling | 3/86 | 81/17913 | 0.033655 | 3 |
| BP | GO:0015696 | ammonium transport | 3/86 | 81/17913 | 0.033655 | 3 |
| BP | GO:0030510 | regulation of BMP signaling pathway | 3/86 | 81/17913 | 0.033655 | 3 |
| BP | GO:0032755 | positive regulation of interleukin-6 production | 3/86 | 81/17913 | 0.033655 | 3 |
| BP | GO:0046849 | bone remodeling | 3/86 | 81/17913 | 0.033655 | 3 |
| BP | GO:0051604 | protein maturation | 6/86 | 353/17913 | 0.03392 | 6 |
| BP | GO:0045445 | myoblast differentiation | 3/86 | 82/17913 | 0.03459 | 3 |
| MF | GO:0002020 | protease binding | 4/84 | 121/16969 | 0.034617 | 4 |
| BP | GO:0002063 | chondrocyte development | 2/86 | 27/17913 | 0.035187 | 2 |
| BP | GO:0046640 | regulation of alpha-beta T cell proliferation | 2/86 | 27/17913 | 0.035187 | 2 |
| BP | GO:1902624 | positive regulation of neutrophil migration | 2/86 | 27/17913 | 0.035187 | 2 |
| BP | GO:0060326 | cell chemotaxis | 5/86 | 253/17913 | 0.035265 | 5 |
| BP | GO:0030838 | positive regulation of actin filament polymerization | 3/86 | 84/17913 | 0.036375 | 3 |
| BP | GO:0019932 | second-messenger-mediated signaling | 6/86 | 360/17913 | 0.036375 | 6 |
| MF | GO:0005518 | collagen binding | 3/84 | 61/16969 | 0.036473 | 3 |
| BP | GO:0032814 | regulation of natural killer cell activation | 2/86 | 28/17913 | 0.036838 | 2 |
| BP | GO:0042533 | tumor necrosis factor biosynthetic process | 2/86 | 28/17913 | 0.036838 | 2 |
| BP | GO:0042534 | regulation of tumor necrosis factor biosynthetic process | 2/86 | 28/17913 | 0.036838 | 2 |
| BP | GO:0070229 | negative regulation of lymphocyte apoptotic process | 2/86 | 28/17913 | 0.036838 | 2 |
| BP | GO:2000406 | positive regulation of T cell migration | 2/86 | 28/17913 | 0.036838 | 2 |
| BP | GO:0060485 | mesenchyme development | 5/86 | 258/17913 | 0.037259 | 5 |

GO：Gene Ontology. TME: Tumor Microenvironment. DEGs: Differentially Expressed Genes. BP: Biological Process. CC: Cellular Component. MF: Molecular Function.
