# Supplementary material for: Identification of prognostic gene signature associated with microenvironment of lung adenocarcinoma
Source: PeerJ. 2019 Nov 29;7:e8128. doi: 10.7717/peerj.8128 (PMC6886493; doi:10.7717/peerj.8128)
Supplement: Supplemental Information 3 [file peerj-07-8128-s003.docx]

The KEGG analysis of TME-related DEGs.

| ID | Description | GeneRatio | BgRatio | FDR | Count |
| --- | --- | --- | --- | --- | --- |
| hsa04611 | Platelet activation | 6/61 | 124/7925 | 0.031306 | 6 |
| hsa05150 | Staphylococcus aureus infection | 5/61 | 96/7925 | 0.031306 | 5 |
| hsa04145 | Phagosome | 6/61 | 152/7925 | 0.031306 | 6 |
| hsa04670 | Leukocyte transendothelial migration | 5/61 | 112/7925 | 0.036546 | 5 |

KEGG: Kyoto Encyclopedia of Genes and Genomes. TME: Tumor Microenvironment. DEGs: Differentially Expressed Genes.
